# Phonon confinement and particle size effect on the low-frequency Raman mode of aurivillius phase Bi_4_Ti_3_O_12_ powders†

**DOI:** 10.1039/d2ra06297f

**Published:** 2023-02-08

**Authors:** Ifeanyichukwu C. Amaechi, Andreas Ruediger, Alain Pignolet

**Affiliations:** a Institut National de la Recherche Scientifique, Centre Énergie, Matériaux & Télécommunications 1650 Boulevard Lionel-Boulet, Varennes Québec J3X 1P7 Canada Ifeanyichukwu.Amaechi@inrs.ca Alain.Pignolet@inrs.ca

## Abstract

We report the systematic measurements in bismuth titanate powders of Raman frequency shift, *ω* and full width at half maximum (FWHM), *Γ* of optical phonons at *q* = 0 obtained between ∼300 K and 673 K in air. Both the particle size and phonon confinement effects are reasonably satisfactory to explain the Raman peak shift and asymmetric broadening observed in the ferroelectric soft phonon mode at 42 cm^−1^. It is shown that the lattice parameter varies as particle size *x*, and its contribution to size-dependent Raman shift and broadening of linewidth follows *ω* ∝ *x*^−0.73^ and *Γ* ∝ *x*^−0.38^ law, respectively. Moreover, a single phonon coupling term corresponding to a three-phonon anharmonic process is sufficient to describe the phonon coupling decay culminating from the softening of this strongly overdamped phonon mode.

## Abstract 2

Both Raman shift, *ω* ∝ *x*^−0.73^, and linewidth, *Γ* ∝ *x*^−0.38^, exhibit a power law dependence on the particle size. The particle size-dependent lattice constant contributed to satisfactory explanation of the Raman shift.

## Graphical abstract

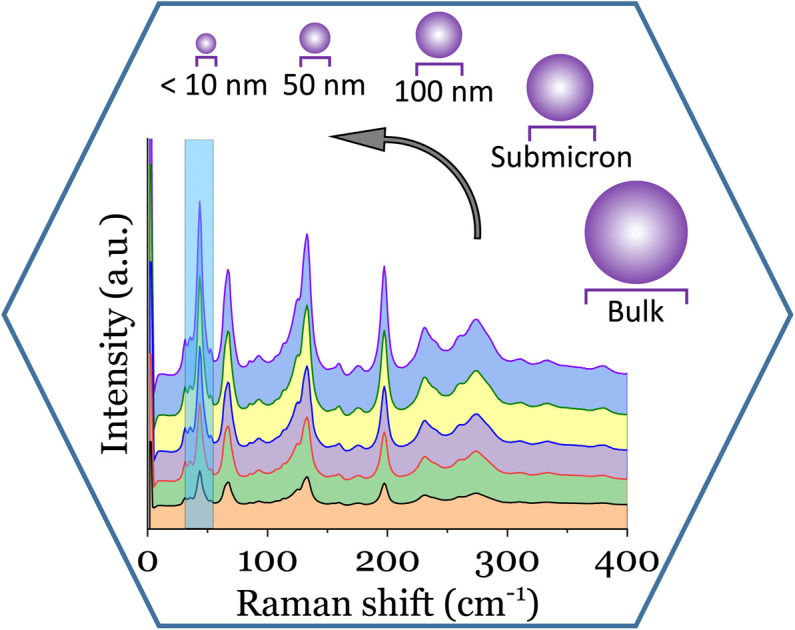

## 1 Introduction

Aurivillius-structure layered bismuth compounds, in particular bismuth titanate, Bi_4_Ti_3_O_12_ have been found to be useful in various applications such as catalysts for hydrogen generation and degradation of contaminants,^1^ positive temperature coefficient resistance (PTCR) heating elements,^2^ lead free piezoelectric transducers, actuators and sensors,^3^ non-volatile random access memories^4,5^*etc.* As a member of the Aurivillius phase family with general formula Bi_2_A_*m*−1_B_*m*_O_3*m*+3_ where A = Bi, B = Ti and *m* = 3, it has a (Bi_2_Ti_3_O_10_)^2−^ triple pseudo-perovskite layer sandwiched between (Bi_2_O_2_)^2+^ layers. Bi_4_Ti_3_O_12_ is polar and can either crystallise in the high-temperature paraelectric tetragonal phase, or the ferroelectric orthorhombic phase. In the orthorhombic phase, it exhibits some unique properties that often depend on particle size. The effect of the particle size on the crystal structure and even phase transition has been investigated extensively using techniques such as X-ray diffraction (XRD) and Raman spectroscopy.^6–8^ The latter is very sensitive to atomic vibrations and local symmetry, thus allowing subtle changes in the microstructure to be easily detected. In terms of Raman selection rules, the orthorhombic phase belonging to the *mmm* Laue class permits only 24 Raman modes (6A_g_ + 2B_1g_ + 8B_2g_ + 8B_3g_). However, experimentally acquired Raman spectra unexpectedly exhibit a smaller number of peaks, as some active Raman modes are weak and sometimes overlap due to degeneracy of some modes.^9^

Previous studies^6,8–10^ on Raman spectroscopy of Bi_4_Ti_3_O_12_ nanoparticles have illustrated how the Raman peak and FWHM are affected when the particle size is decreased. Such redshift in peak energy and broadening have been related to the effect of charge transfer in the Bi–O–Ti system instead of internal strain associated with the particles or particle size effect.^8^ At this point, it is worth mentioning that several factors such as strain, broadening due to size distribution, the presence of defects, phonon confinement *etc.* can all contribute to the subtle changes in the features of the Raman spectra.^11^ While size measurement employing Raman spectroscopy is predicated on the aforesaid dependences, the explanation of these dependences using any of the semi-empirical models such as the phonon-confinement model (PCM) is lacking. For instance, ref. 6 and 10 explained the smearing out of the Raman modes in terms of progressive decrease in particle size, thus predicting a critical size below which ferroelectric ordering in the material disappears. It is therefore important to examine the effect of particle size on the phonon–phonon coupling in the orthorhombic Bi_4_Ti_3_O_12_ particles.

In this study, we report the preparation of controlled sizes of single-phase Bi_4_Ti_3_O_12_ nanoparticles through a scalable microwave-assisted hydrothermal method and subsequent thermal treatments; and using the existence of both strain, particle size effect and phonon confinement, the peak shift of the soft phonon mode can be explained satisfactorily. By combining the structural analysis employing XRD with Raman scattering technique, we further provide an explanation for the temperature-dependent variation of the Raman frequency and FWHM as a function particle size given that the measure of the decay of optical phonons with those of acoustic phonons differs for the same particle size.

## 2 Materials and methods

Various particle sizes of Bi_4_Ti_3_O_12_ nanocrystals are synthesized by microwave-assisted hydrothermal method using bismuth nitrate, Bi(NO_3_)_3_·5H_2_O and titanium butoxide, Ti(C_4_H_9_O)_4_ as the sources of Bi^3+^ and Ti^4+^, respectively, followed by a thermal treatment. By maintaining a Bi/Ti mole ratio of 1.33, Ti-butoxide is dissolved in 5 mL of ethanol under constant stirring, labelled A while bismuth salt solution is prepared by dissolving corresponding stoichiometric quantity of Bi(NO_3_)_3_·5H_2_O in 5 mL of glacial acetic acid, labelled B. Subsequently, 2 mL of aqueous ammonia (28%) as a chelating agent is then added into A dropwise. After 15 min of vigorous stirring, solution B (and varying amount of polyethylene glycol (PEG-8000) in the case of small particle sizes) is added to A gradually and then stirred further for 20 min before transferring the homogeneous mixture to a 23 mL capacity polytetrafluoroethylene-based Teflon container. This is then sealed in a polymer autoclave (from Parr Instrument) and subjected to microwave heating using a Paderno microwave oven (2.45 GHz) with inverter technology. After the microwave exposure using power level, P1 for 10 min and cooling down to ambient temperature, the content is then washed with both ethanol and distilled water several times before drying in the oven at 80 °C for 10 h. A thermal treatment at 700 °C for 2 h is thereafter performed to crystallise Bi_4_Ti_3_O_12_. Larger Bi_4_Ti_3_O_12_ particles were obtained by sintering the as-synthesized nanoparticles. All thermal treatments are done using a heating rate of 20 °C min^−1^.

The phase identification is done using XRD (Bruker D8 Advance diffractometer) with Cu Kα radiation (*λ* = 1.5406 Å). Knowing the crystallography of Bi_4_Ti_3_O_12_ and by means of fitting the X-ray diffraction peak position the lattice parameters were determined. Bright field images of the samples were acquired using a JEOL JEM-2100F transmission electron microscope. The powdered samples were ultrasonically dispersed in methanol prior to dip-coating unto a Lacey carbon-coated Cu grids (LC400-Cu). Raman scattering measurement was performed with a Horiba iHR320 system equipped with a thermoelectrically cooled Horiba Scientific Synapse Back-Illuminated Deep Depletion CCD detector. The excitation source was a 473 nm solid-state blue Cobolt 04-01 laser operating at 0.75 mW. All the temperature-dependent Raman measurements were done with a Linkam THMS600 stage, in which case a heating/cooling rate of 10 °C min^−1^ has been used to achieve the set temperature. Prior to acquisition of any Raman spectrum, a time interval of 10 min was given to allow the sample on the Linkam stage to reach thermal equilibrium.

## 3 Results and discussion

**Fig. 1:**
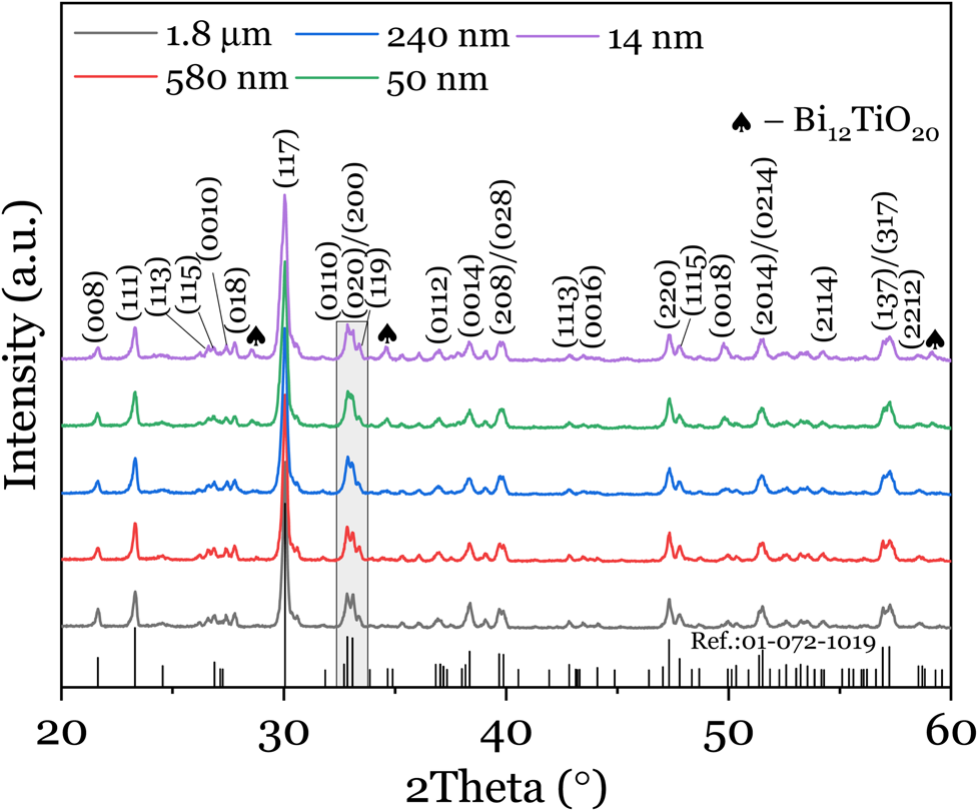
XRD patterns of as-synthesized and sintered Bi_4_Ti_3_O_12_ particles of different sizes.

X-ray diffraction patterns of the synthesized Bi_4_Ti_3_O_12_ powders are displayed for different sizes in Fig. 1. All the powders are polycrystalline and indexed to the single orthorhombic phase of Bi_4_Ti_3_O_12_ with the space group *B*2*ab*, consistent with the reference pattern, PDF 01-072-1019.^12^ The growth kinetics usually follows two routes: (i) the direct route; in which Bi_2_O_3_ reacts with TiO_2_ to produce Bi_4_Ti_3_O_12_*i.e.* 2Bi_2_O_3_ + 3TiO_2_ → Bi_4_Ti_3_O_12_, and (ii) an indirect route; in which case an intermediate phase of Bi_12_TiO_20_ is formed prior to a complete transformation at elevated temperature *i.e.* Bi_12_TiO_20_ + 8TiO_2_ → 3Bi_4_Ti_3_O_12_.^13^ For the 14 nm and 50 nm, the peak around 28.5° and 34.6° are attributes of this phase ascribable to the lowered calcination temperature (<700 °C). This suggests that complete crystallisation of the orthorhombic phase is favored at high temperature. Regardless of this barely detectable secondary phase, all the samples exhibit good orthorhombicity (often evaluated by the ratio of the lattice parameters, *a*/*b*) as evidenced by the peak splitting into (200) and (020) planes at 2*θ* = 33° (Fig. S1a†). The plot, *a*/*b vs.* particle size is also shown in Fig. S1b† of which a critical particle size of 29 ± 10 nm is estimated, consistent with the literature.^14^ We then evaluated the dependence of the lattice parameter, *a* on particle size as this potentially affects the position of the Raman peak (which will be discussed next). The choice of the lattice parameter *a* stems from the fact that spontaneous polarization in ferroelectric bismuth-containing layer-structured perovskites is much larger along the *a*-axis compared to *c*-axis^15^ and as such would impact the Raman lineshape. As shown in Fig. S2,† it decreases with the particle size *x*^7^ and a fitting function of the form, *a*(*x*) = *a*_0_ + *k*/*x* is used to fit the experimental data, where *a*_0_ is the bulk lattice parameter, 5.445 ± 0.003 Å and *k* is a constant, 0.647 ± 0.152 Å^2^ determined through curve fitting. While the TEM images are mainly characterised by ellipsoidal, elongated fibrous networks and a certain degree of agglomeration, the mean particle sizes were rather estimated from histograms, which are plotted by analysing the size of the particles from the acquired TEM images (Fig. S3† and insets). The size distribution is nearly Gaussian, 
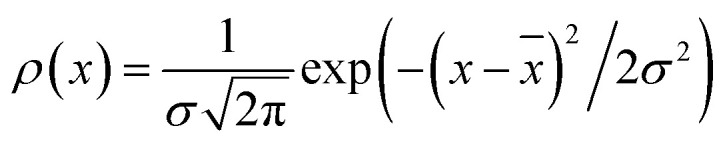
, with *x̄* and *σ* as the mean particle size and standard deviation, respectively as determined from the histogram. The post-synthesis conditions, lattice constant and mean particle size are summarised in Table 1.

**Table 1:** The post-synthesis conditions, lattice parameter and mean particle size of the samples

Calcination temperature (°C)/2 h	Sintering temperature (°C)	Lattice parameter, *a* (Å)	Mean particle size, *x* ± *σ* (nm)
500	—	5.4048 ± 0.002	14 ± 3
600	—	5.4208 ± 0.001	50 ± 9
700	—	5.4329 ± 0.001	240 ± 66
700	850, 1 h	5.4453 ± 0.001	580 ± 116
700	950, 6 h	5.4480 ± 0.001	1800 ± 443

**Fig. 2:**
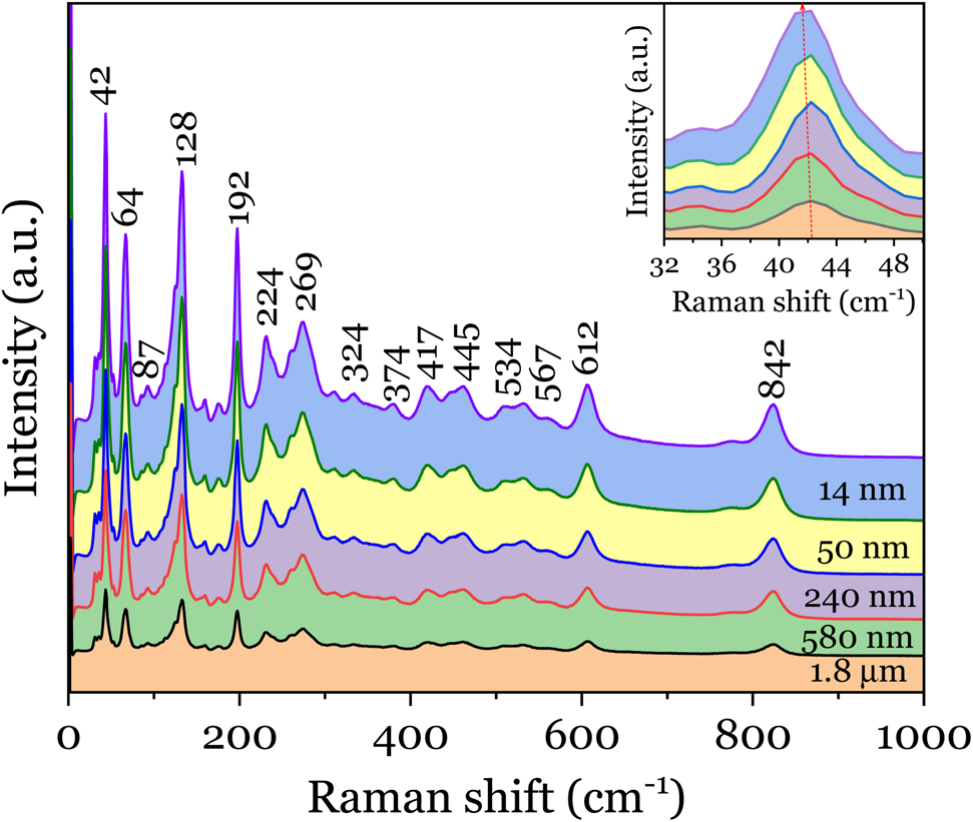
Room temperature Raman spectra of the Bi_4_Ti_3_O_12_ powders. Inset is the low-frequency region of the spectra with emphasis on the 42 cm^−1^ soft phonon mode.

Raman spectroscopy is further deployed to study the lattice dynamics of the micro- and nano-crystalline Bi_4_Ti_3_O_12_ powders. The complete Raman spectrum of the different powders acquired at room temperature is shown in Fig. 2. The spectra show some intense Raman peaks resulting from strong ionic interactions, mainly coming from the octahedrally coordinated Ti–O bonds as well as between the pseudo-perovskite block of ocathedra and the Bi–O bonded rigid bilayer.^16^ The phonon assignments are done by comparing Raman spectra of Bi_4_Ti_3_O_12_ from previously published data.^10,16–18^ The internal vibrations of the triply connected TiO_6_ octahedra are reflected by 224, 269, 324, 534, 567 and 841 cm^−1^ modes whereas the intense 64 and 128 cm^−1^ modes denote the motion of the (Bi_2_O_2_)^2+^ layers with respect to (Bi_2_Ti_3_O_10_)^2−^ slab, which is invariably prevalent to Bi-layered perovskites.^18^ The observation of the modes associated to the rigid bilayer and TiO_6_ in pseudo-perovskite structure is an indication that the Bi_4_Ti_3_O_12_ is well crystallised. Due to high degree of freedom available for the TiO_6_ octahedron, vibrations such as symmetric stretching, bending, torsional or even combination of any of those are possible. In terms of mode degeneracy, the 567, 612 and 841 cm^−1^ exhibit the A_1g_ character; 324 and 445 cm^−1^ have the characteristics of B_1g_, and 224, 269 and 534 cm^−1^ have the B_2g_ + B_3g_ behaviour.^17^ The 224 and 269 cm^−1^ modes are known to be connected to orthorhombic distortion of the octahedra and also have the capacity to lift the doubly *E*_g_ degeneracy.^17^ The splitting and further broadening of the original 269 cm^−1^ mode indicate a symmetric bending of O–Ti–O associated to the displacement of Ti^4+^ in the octahedra. This corroborates the large orthorhombic splitting observed in XRD patterns at 2*θ* = 33°. For the purpose of clarity, the low-frequency region with emphasis on the 42 cm^−1^ mode is shown in the inset of Fig. 2 since the peak at 42 cm^−1^ is the Raman mode where the most significant effect of confinement could be observed. Indeed, the Raman spectra of this mode reveal a size dependence of the Raman shift. Expectedly, the peak redshifts and broadens asymmetrically as the particle size decreases. This implies an increased weight of the off-centre phonons. Such asymmetric broadening in the Raman lineshape is best described within the framework of PCM, which correlates the subtle changes with particle size. In principle, a Gaussian weighting function is typically used to model the Raman line profile in which the resulting frequency-dependent Raman intensity, *I*(*ω*) is expressed by^19–21^1
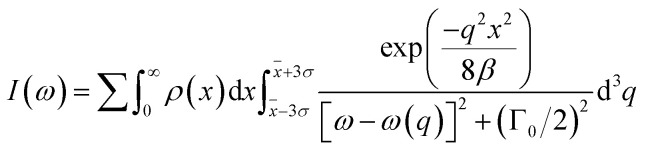


**Fig. 3:**
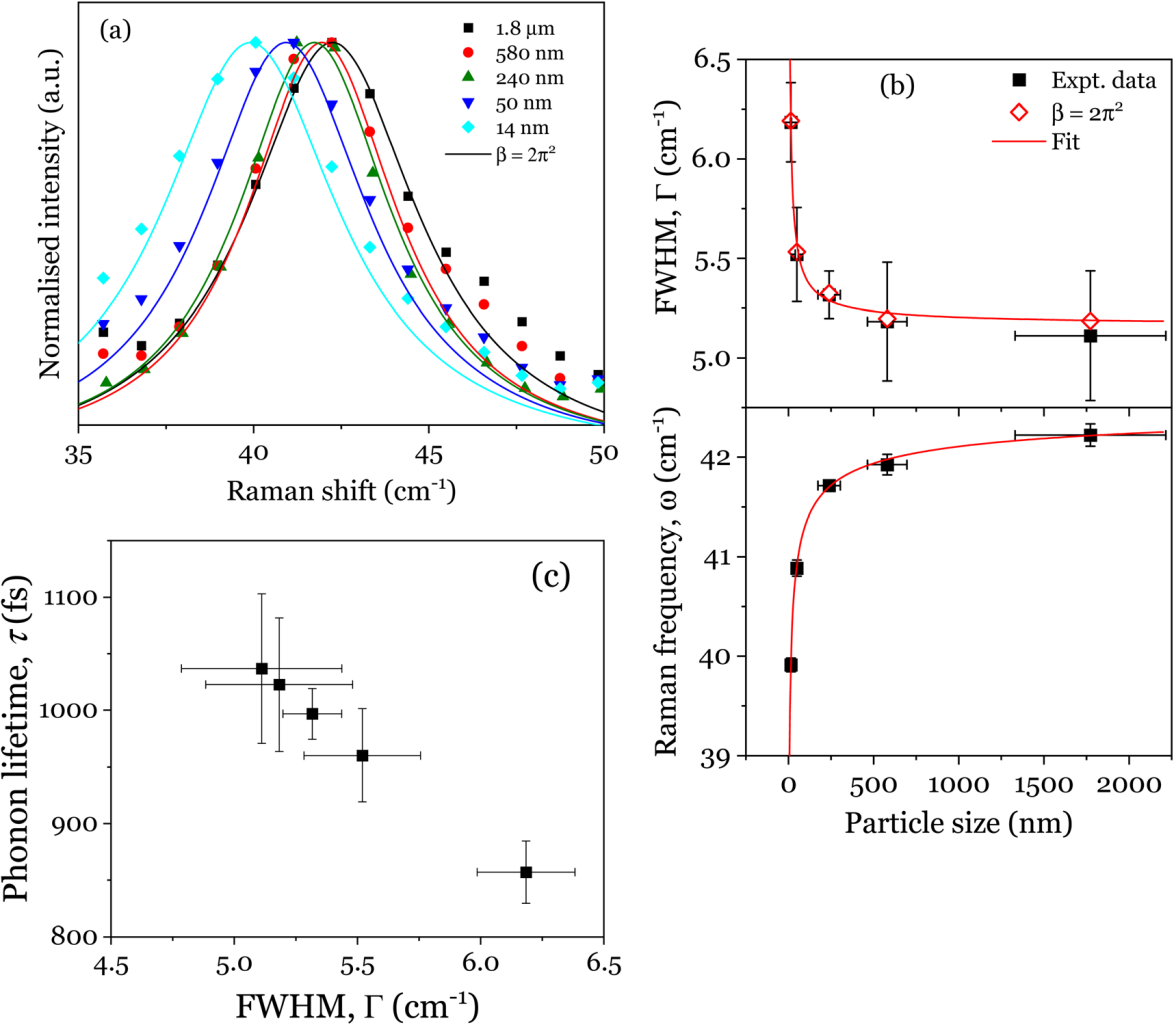
(a) Raman spectra and PCM fitting using eqn (1). Solid lines are fitted curves. (b) Experimentally measured Raman shift (lower) and corresponding FWHM (upper) as a function of particle size, together with their fitted curves. The open diamonds denote the results obtained using the Campbell-based PCM. (c) Variation of phonon lifetime with FWHM.

The inclusion of the particle size distribution, *ρ*(*x*) experimentally measured yields an excellent fit, allowing a 99.5% confidence interval to be chosen as the integral limits. In eqn (1), *q* is the wave vector defined by 2π/*a*, where *a* is the lattice constant, *β* is the confinement factor, which assumes the value of either 1 according to the model of Richter *et al.*^20^ or 2π^2^ by the generalized Campbell and Fauchet model (*i.e.* the contribution of phonons away from the zone centre is considered),^19^*Γ*_0_ is the FWHM, and *ω*(*q*) is the phonon dispersion of the 42 cm^−1^ mode^22^ fitted with a parabolic function, *ω*(*q*) = *A* + *Bq* + *Cq*^2^. Since no data for *Γ*_0_ has been reported, it was rather estimated by fitting the 42 cm^−1^ mode in the Raman spectrum of the powder sintered at 950 °C. Moreover, the Bi_4_Ti_3_O_12_ nanoparticles being a powdered material allows the infinitesimal volume, d^3^*q* around the Brillouin zone in the PCM to be replaced by 4π*q*^2^d*q*. As the distribution of the particle size is nearly normal (refer to the histogram plots in inset of Fig. S3†) and will introduce some kind of broadening in the Raman lineshape, eqn (1) is therefore integrated over this Gaussian size distribution in order to obtain the Raman intensity, *I*(*ω*). Expectedly, as shown in Fig. 3a both the effect of particle size and phonon confinement lead to asymmetry with a corresponding shift to lower wavenumber. The size-dependent FWHMs calculated by Campbell model (*β* = 2*π*^2^) are quite comparable to the experimental data (see Fig. 3b). To further describe the Raman shift arising from the confinement effect, a confinement model of the form^23^ is used given the lattice constant dependence on the particle size observed and described above (Fig S2†). *ω*(*x*) and *Γ*(*x*) are Raman frequency and FWHM with size, *x* while *ω*_0_ and *Γ*_0_ are the frequency and FWHM of the optical phonon at the zone center. *X*, *Y*, *γ* and *γ*′ are parameters which define the confinement arising from finite size. By fitting eqn (2) to the experimental data of Fig. 3b, we obtain *ω*_0_ = 5.08 ± 0.002 cm^−1^, *X* = 11.85 ± 0.18 cm^−1^, *γ* = 0.73 ± 0.005 and *Γ*_0_ = 42.67 ± 0.14 cm^−1^, *Y* = 9.83 ± 0.81 cm^−1^, *γ*′ = 0.38 ± 0.038. The fitted results for *ω*_0_ and *Γ*_0_: 5.08 cm^−1^ and 42.67 cm^−1^, are in good agreement with those extracted for the bulk powder sintered at 950 °C. The relationship between Raman shift and FWHM with particle size follow a *x*^−0.73^ and *x*^−0.38^ law, respectively. The curve fitting also revealed a finite size of ∼29 nm below which the shape of the Brillouin zone and details of the dispersion curve is expected to become critical.^24^ The linewidths were further analysed in order to estimate the phonon lifetime based on the energy-time uncertainty relation, *τ* = *ℏ*/Δ*E*.^25^ Using the aforementioned relation, where *ℏ* and Δ*E* are the reduced Planck's constant (5.3 × 10^−12^ cm^−1^ s) and FWHM of the individual lineshapes, the phonon lifetimes calculated are in the range 850–1100 fs as shown in Fig. 3c. The plot shows the shortening of phonon lifetime associated to mainly size effect. However, a length scale of ∼4 nm assuming an isotropic speed of sound in Bi_4_Ti_3_O_12_ is 4.79 km s^−1^(ref. 26) appears to be underestimated, and thus the effect of phonon confinement even presumed to set in prematurely in the as-synthesized powder. Such discrepancy could be due to two or more competing phonon lifetime-shortening mechanisms in the crystal; one of which could be phonon scattering at defect sites. Non-stoichiometry in materials in the form of oxygen vacancy contributes to the asymmetric broadening observed in Raman lineshapes especially for low-frequency (soft) modes,^11,27^ although the effect of the oxygen vacancies is usually less pronounced compared to those of phonon confinement, size distribution and strain^11^. The presence of point defects is pervasive in metal oxides and so it will be as well for layered structured oxides such as bismuth titanate. However, this issue is not addressed here due to the fact that a quantification of concentration of the defects leading to a reasonable interpretation of Raman data is somewhat complicated as there should be a prior knowledge of the full dynamical matrix of the material in order to be able to estimate it.^28^2
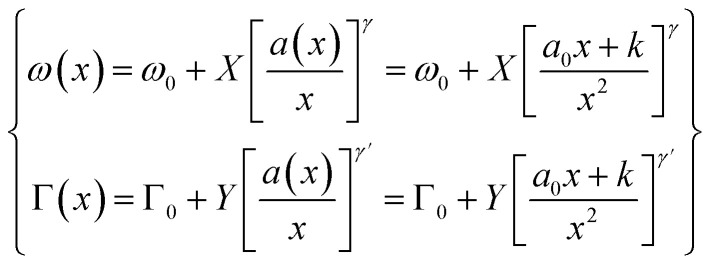


**Fig. 4:**
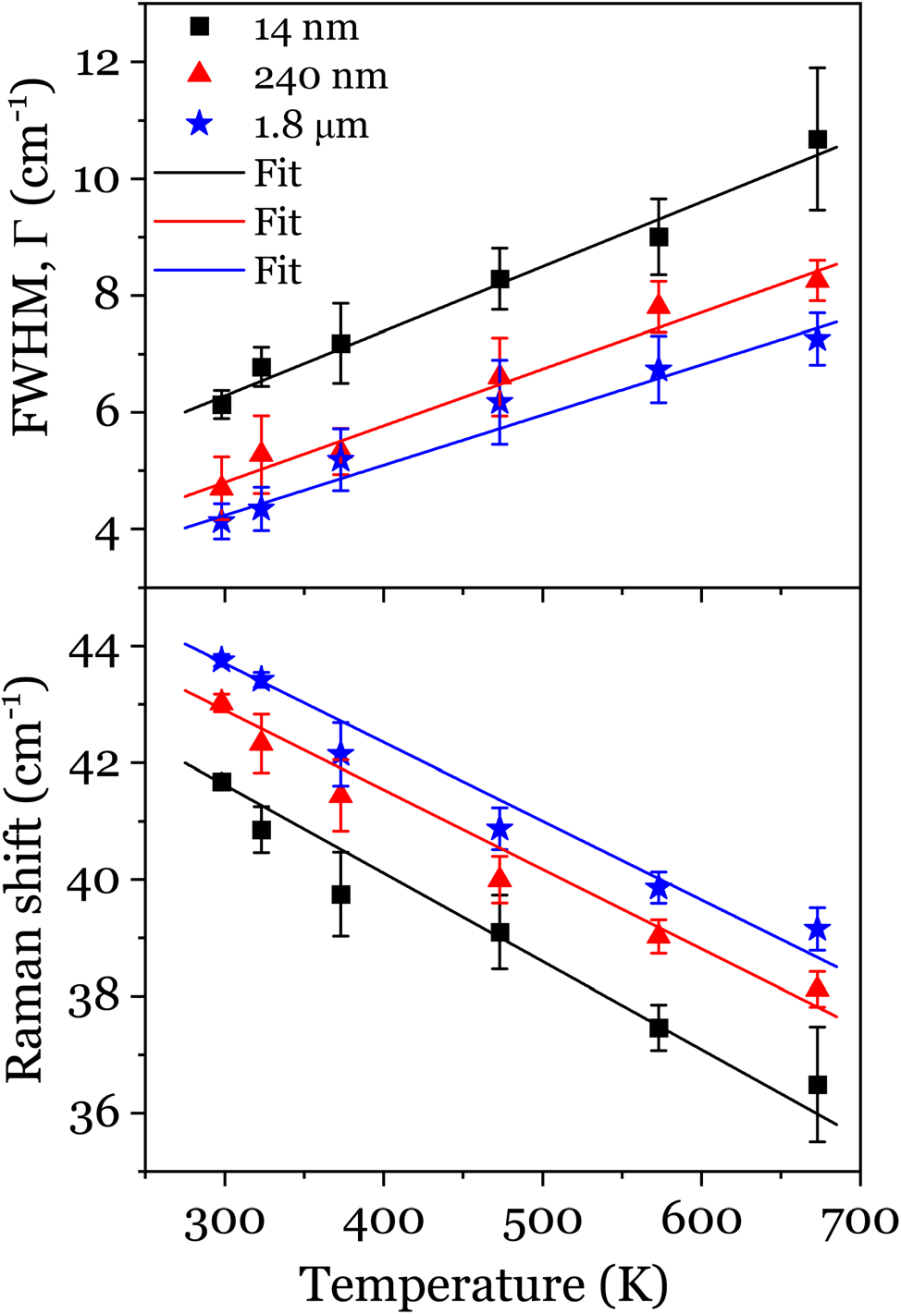
The variation of Raman shift (lower), and FWHM (upper) with temperature. The solid lines are fits based on eqn (3a) and (3b), respectively.

The temperature-dependence of the Raman spectra of the 14 nm, 240 nm and 1.8 μm Bi_4_Ti_3_O_12_ powders has also been investigated (Fig. S4†) after correcting with the Bose-Einstein occupation factor, *I*_R_(*ω*) = *I*(*ω*)/[*n*(*ω*) + 1]^29^ and applying Rayleigh line correction. 
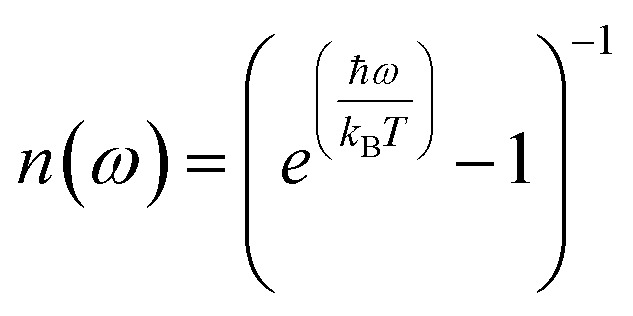
, where *ℏ*, *k*_B_ and *T* are the Planck's constant, Boltzmann constant and temperature, respectively. In Fig. S4,† the soft mode at 42 cm^−1^ is strongly dependent on temperature with an asymmetric broadening and continuous shift to the lower wavenumber up to ∼4 cm^−1^. While Bi_4_Ti_3_O_12_ undergoes a displacive-type transition and the ferroelectric soft phonon mode is affected markedly, we note that they are not completely smeared out at 573 K. In fact, the strong temperature dependence is associated to the anharmoncity in Bi–O bonds, which significantly softens the phonon mode.^30^ Since this mode is responsible for the existence of spontaneous polarisation in Bi_4_Ti_3_O_12_, it implies that a ferroelectric phase transition has not yet occurred; although it has been speculated that a premature phase transition is probable at a lower temperature.^27^ However, the progressive diminution of the intensity of these modes attests to the spontaneity of the process. Although the intensities of the rigid layer (64 cm^−1^) and TiO_6_-related Raman modes (269 and 841 cm^−1^) are nearly unaffected and quasi-independent of temperature, they are, however, all equally accompanied by some degree of broadening. The temperature dependence of the 42 cm^−1^ peak position and FWHM from 298 K to 673 K are displayed Fig. 4. For all the powders, an increase in temperature results in a decrease and increase in the peak position and FWHM, respectively. There is also no significant distinction between the Raman parameters of the 240 nm and 1.8 μm powders. In other words, the submicron 240 nm powder can be regarded as bulk to some extent. For the 14 nm powder, there is a clear difference especially for the linewidth. Although the Linkam stage placed a limitation in terms of reaching higher temperatures, Yu *et al.*^31^ have demonstrated that the Curie temperature, *T*_C_ of different sizes of Bi-layered powders can easily be estimated by fitting the soft mode using a simple damped harmonic oscillator model. In the event of low and high temperature regimes, the anharmonic coupling between optical phonons is totally different. For instance, an optical phonon can couple to two lower energy phonons giving rise to a three-phonon process, ∼*ℏω*_0_/2*k*_B_*T*, and a four-phonon process *i.e.* a phonon coupling to three lower energy phonons, ∼*ℏω*_0_/3*k*_B_*T* can also be obtained in similar fashion.^32^ The former is proportional to *T* while the latter is proportional to *T*^2^ at moderate and higher temperatures, respectively. A more generalized model to describe the phonon–phonon coupling culminating to the Raman frequency shift and FWHM broadening in the absence of thermal expansion is given by eqn (3)^11,32^3a
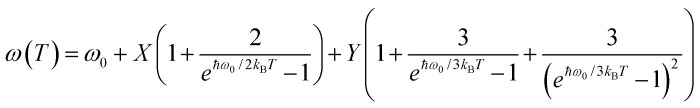
3b
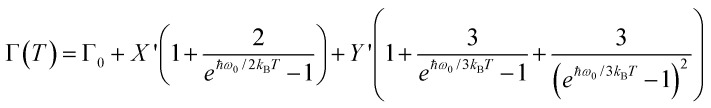
where *ω*_0_ is Raman frequency, *Γ*_0_ is the FWHM independent of phononic thermal population,^11^*X*/*X*′ and *Y*/*Y*′ are prefactors of the second and third term, denoting the contribution of three- and four-phonon processes, respectively to the shift of Raman peak and asymmetry. In the high temperature regime, the third term in eqn (3) obviously has no contribution and can be disregarded as it strongly depends on *T*^2^. In other words, setting *Y* to zero in the fitting function will not affect the curve. Hence by method of least-squares, eqn (3a) is fitted to the experimental data of Fig. 4. Similarly, the FWHM as a function of temperature is then fitted using the values of *ω*_0_ obtained from eqn (3a) and setting *Y*′ to zero. Both the temperature-independent (*ω*_0_ and *Γ*_0_), and temperature-dependent (*X* and *X*′) fit parameters are listed in Table 2. It is observed that by decreasing the particle size, the parameter *ω*_0_ decreased from 48 cm^−1^ to 45 cm^−1^ as a consequence of the phonon confinement effect. A similar observation was even reported for a triply degenerate first-order Raman mode of oxygen-deficient cerium oxide nanoparticles.^11^ The magnitude of the temperature-independent FWHM, *Γ*_0_ appears to be comparable for the bulk (1.7 ± 0.3 cm^−1^) and 240 nm (1.9 ± 0.4 cm^−1^) powders, thus confirming the nondescript nature of the two powders. Also by decreasing the particle size to 14 nm, the asymmetry is seen to increase by 1.1 cm^−1^ (roughly 65% of that of the bulk value). On the account of the effect of phonon coupling defined by the term *X*′, there is a slight increase in asymmetry, although not necessarily significant and sufficient to explain the increase observed. Such a trend has been reportedly attributed to a faster decay of phonons in nanoparticles in comparison to the bulk.^33^

**Table 2:** The fit parameters for Raman peak shift and FWHM as estimated from eqn (3); obtained by setting both *Y* and *Y*′ to zero

Particle size	*ω*_0_ (cm^−1^)	*X*	*Γ*_0_ (cm^−1^)	*X*′
14 ± 3 nm	45.2 ± 0.3	−0.35 ± 0.03	3.0 ± 0.4	0.25 ± 0.02
240 ± 66 nm	47.0 ± 0.4	−0.32 ± 0.02	1.9 ± 0.4	0.23 ± 0.02
1.8 ± 0.4 μm	47.8 ± 0.3	−0.25 ± 0.02	1.7 ± 0.3	0.21 ± 0.02

## 4 Conclusion

In summary, Bi_4_Ti_3_O_12_ powders were synthesised by microwave-assisted hydrothermal method followed by thermal treatment between 500 °C and 700 °C. The growth kinetics is seen to favour the direct transformation of hydrolysis of titanium dioxide and bismuth oxide to bismuth titanate powders at high temperature rather than lower temperatures where the undesired phase of Bi_12_TiO_20_ dominates instead. While the XRD confirmed not only the stable but dominant orthorhombic phase even for the smallest particles, the *in situ* Raman spectroscopic investigation of these powders carried out at temperatures between 300 K and 675 K shows a marked softening of the lowest mode. Although, the intensity of this mode does not completely smear out even at 573 K, the thermally induced anharmoncity introduce a redshift in the Raman frequency and broadening of the linewidth. The result obtained through the energy-time uncertainty relation and curve fitting revealed a finite particle size below which both the shape of the Brillouin zone and details of the dispersion curve become critical. Hence, for applications (ferroelectric memory, bulk photovoltaic effect *etc.*) that require the presence of the polar phase at ambient temperature, particle sizes below approximately 29 nm should not be considered. Regardless of the “minor” contribution of defect culminating from non-stoichiometry to Raman lineshapes, the Campbell-based PCM was able to describe the Raman lineshape of these powders taking the particle size distribution and size-dependent lattice constant into account.

## Conflicts of interest

The authors have no conflicts of interest to declare.

## Supplementary Material

RA-013-D2RA06297F-s001
