# Supplementary material for: Phonon confinement and particle size effect on the low-frequency Raman mode of aurivillius phase Bi4Ti3O12 powders
Source: RSC Adv. 2023 Feb 8;13(8):4917–23. doi: 10.1039/d2ra06297f (PMC9906980; doi:10.1039/d2ra06297f)
Supplement: RA-013-D2RA06297F-s001 [file RA-013-D2RA06297F-s001.pdf]

Electronic Supplementary Material (ESI) for RSC Advances.
This journal is © The Royal Society of Chemistry 2023

Phonon confinement and particle size effect on the low-frequency Raman mode of Aurivillius phase $Bi_4Ti_3O_{12}$ powders

Ifeanyichukwu Amaechi, Andreas Ruediger and Alain Pignolet

Institut National de la Recherche Scientifique, Centre Énergie, Matériaux & Télécommunications, 1650 Boulevard Lionel-Boulet, Varennes, Québec, J3X 1P7, Canada.

Corresponding authors: *Ifeanyichukwu.Amaechi@inrs.ca; Alain.Pignolet@inrs.ca*

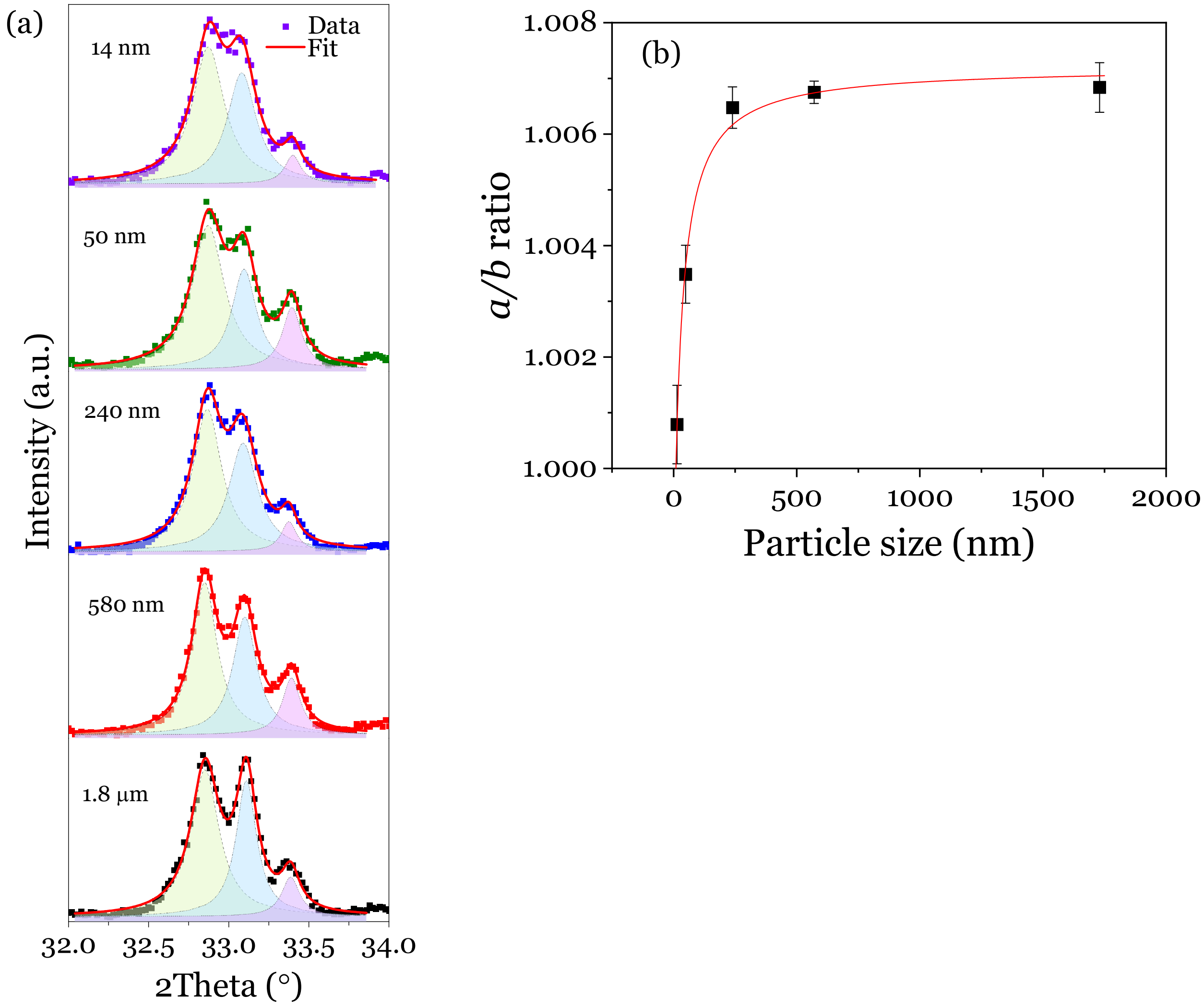

Fig. S1: (a) Expanded view and Lorenztian peak fit of the diffraction line at $2\theta = 33°$; and (b) size dependent orthorhombic distortion (i.e. a/b ratio) vs. particle size x. The experimental data were fitted according to Eq. 2 of Ishikawa et al [1].

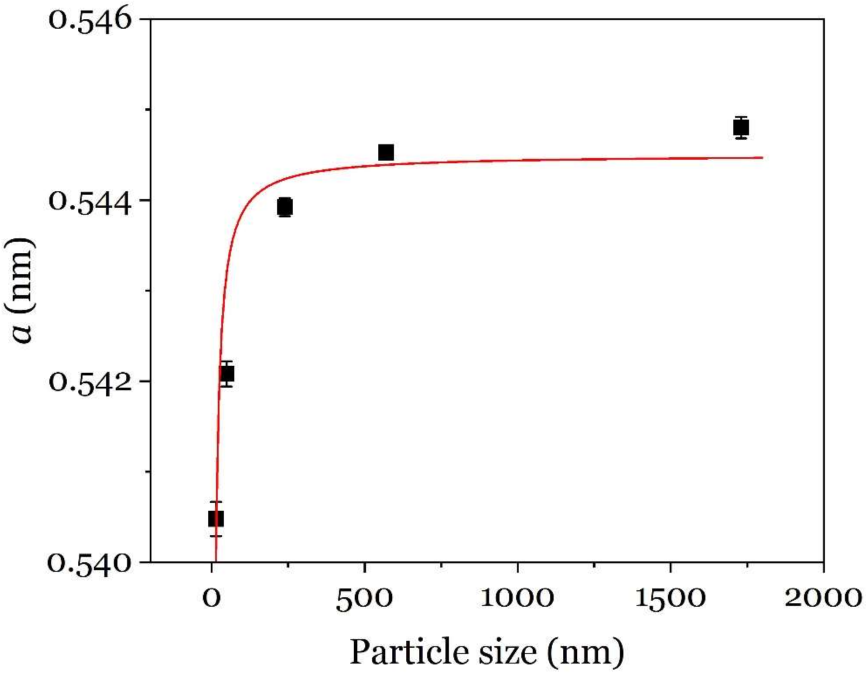

Fig. S2: The variation of the lattice parameter *a* with the particle size.

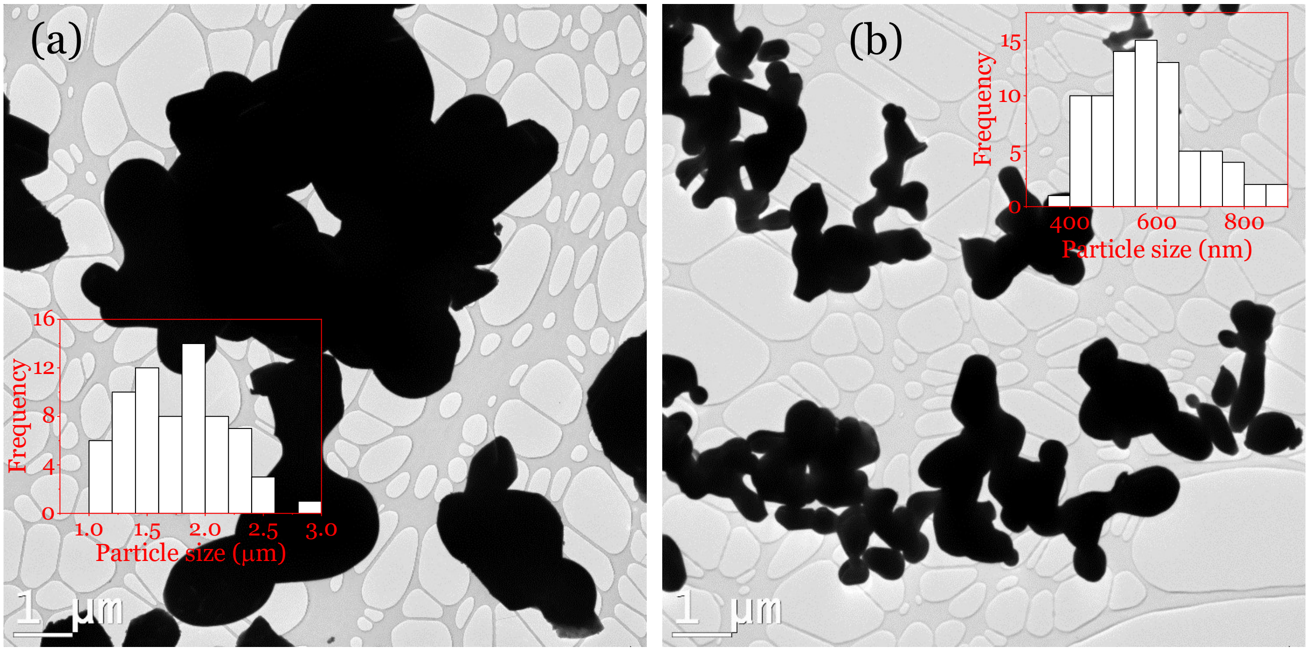

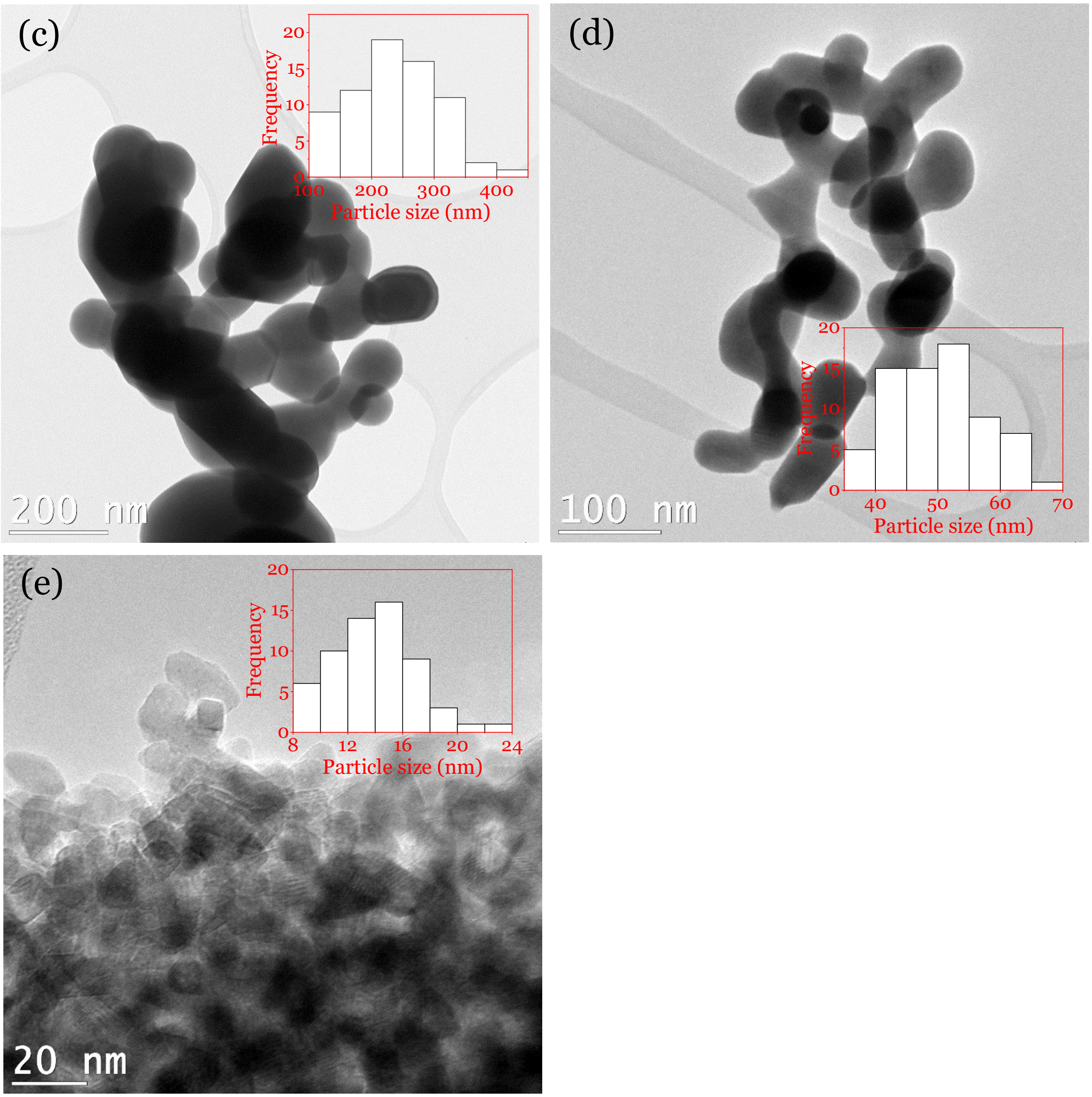

Fig. S3: TEM images of the (a) 1.8 µm (b) 580 nm (c) 240 nm (d) 50 nm and (e) 14 nm bismuth titanate powders. Insets are the histogram reflecting the particle size distribution of the powders.

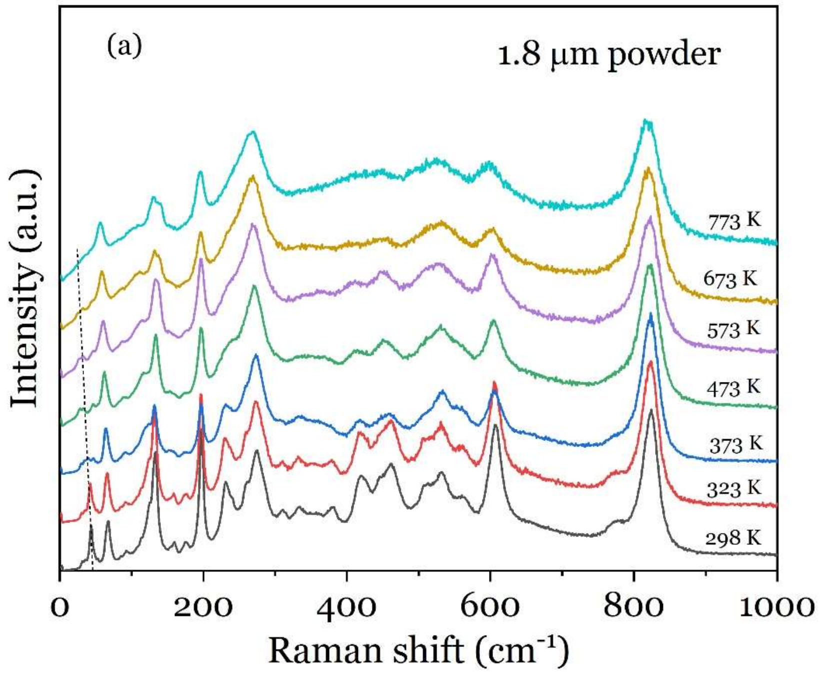

(a)
1.8 μm powder
773 K
673 K
573 K
473 K
373 K
323 K
298 K
Intensity (a.u.)
0
200
400
600
800
1000
Raman shift (cm-1)

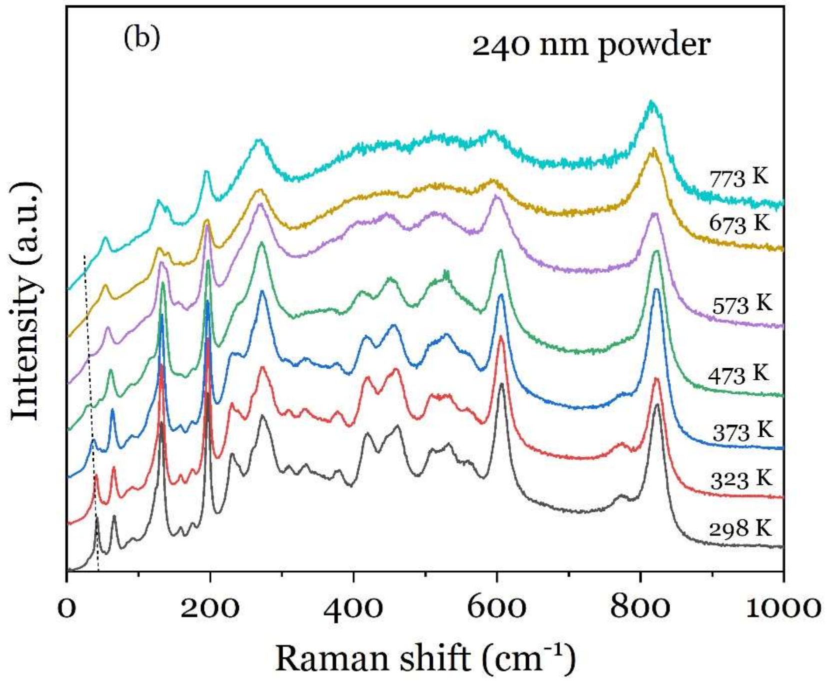

(b)
240 nm powder
773 K
673 K
573 K
473 K
373 K
323 K
298 K
Intensity (a.u.)
0
200
400
600
800
1000
Raman shift (cm-1)

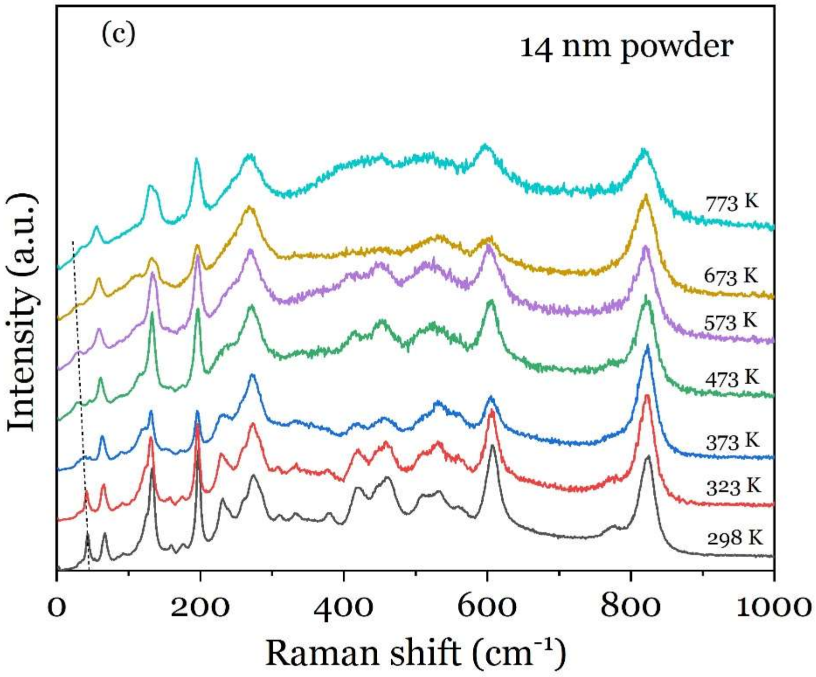

Fig. S4: Raman spectra of the (a) 1.8 μm, (b) 240 nm, and (c) 14 nm bismuth titanate powders as a function of temperature. All the spectra are corrected with the Bose-Einstein occupation factor.

References

[1] K. Ishikawa, T. Nomura, N. Okada, K. Takada, Size effect on the phase transition in $PbTiO_3$ fine particles, Jpn. J. Appl. Phys., 35 (1996) 5196-5198.
